# Effects of Different Adjuvants on the Protective Efficacy of a Subcellular Vaccine Against *Chlamydia abortus* Infection in Sheep

**DOI:** 10.3390/vaccines13060609

**Published:** 2025-06-05

**Authors:** Morag Livingstone, Kevin Aitchison, Javier Palarea-Albaladejo, Sergio Gastón Caspe, Clare Underwood, Holly Hill, Cameron Cunnea, Kelly Stronach, Francesca Chianini, Gary Entrican, Sean Ranjan Wattegedera, David Longbottom

**Affiliations:** 1 Moredun Research Institute, Pentlands Science Park, Penicuik, Midlothian EH26 0PZ, UK; morag.livingstone@moredun.ac.uk (M.L.); kevin.aitchison@moredun.ac.uk (K.A.); gaston.caspe@moredun.ac.uk (S.G.C.); clare.underwood@moredun.ac.uk (C.U.); holly.hill@moredun.ac.uk (H.H.); cameron.cunnea@moredun.ac.uk (C.C.); kjstronach@gmail.com (K.S.); fchianini@yahoo.com (F.C.); gary.entrican@roslin.ed.ac.uk (G.E.); sean.wattegedera@moredun.ac.uk (S.R.W.); 2 Biomathematics and Statistics Scotland, Edinburgh EH9 3FD, UK; javier.palarea@udg.edu

**Keywords:** *Chlamydia abortus*, ovine enzootic abortion, vaccine development, vaccine efficacy, gross placental pathology, quantitative real-time PCR, serological analysis, cytokine analysis

## Abstract

Background/Objective: Recently, we published three studies describing the development and optimization of a new, safe, and efficacious vaccine to protect sheep from ovine enzootic abortion, which is caused by the zoonotic pathogen *Chlamydia abortus*. The vaccine, which can be delivered through a single inoculation, is based on a detergent-extracted outer membrane protein (chlamydial outer membrane complex or COMC) preparation of the pathogen. This study aimed to optimize the vaccine further by comparing the effects of different adjuvants on protective efficacy. Methods: We evaluated the effectiveness of three different vaccines (2.5 µg COMC) formulated with one of three adjuvants (Montanide ISA 70VG, Montanide ISA 61VG, and QuilA) to reduce the rate of abortion, placental load and pathology, and post-partum vaginal shedding of organisms in comparison to our benchmark 20 µg COMC/Montanide ISA 70 VG vaccine and a challenge control group of animals. The humoral and cellular immunological responses to vaccination and to challenge were also assessed. Results: The two low-dose Montanide formulated vaccines resulted in low abortion rates of 3.2 and 8.1% for ISA 70 VG and ISA 61 VG, respectively, which were comparable to the benchmark vaccine group (2.7%) and considerably lower than the QuilA (23.7%) and challenge control (36.8%) groups. Similarly, the Montanide-adjuvanted groups had much lower bacterial loads (range: 136–431 genome copies) on vaginal swabs post-parturition than the QuilA (8.9 × 10^4^ copies) and challenge control (2.4 × 10^5^ copies) groups. Conclusions: The results showed that both Montanide adjuvants are more effective for maximizing COMC vaccine efficacy than the QuilA adjuvant and result in much lower bacterial shedding of the pathogen post-parturition, which is important for minimizing potential transmission to naïve animals.

## 1. Introduction

*Chlamydia abortus* (*C. abortus*) is a Gram-negative obligate intracellular bacterium and is the etiological agent of ovine enzootic abortion (OEA) (syn: enzootic abortion; ovine chlamydiosis; enzootic abortion of ewes (EAE)), which was first described by Stamp and colleagues in 1950 [1]. The disease is a common cause of abortion and fetal loss in sheep and goats in many countries throughout the world [2,3,4] and has a significant economic impact on the livestock industry [5,6]. To a lesser degree, the pathogen can also infect other animal species such as cattle, pigs, and horses [2,7]. Importantly, *C. abortus* is also a zoonotic pathogen and can cause abortion and severe disease in pregnant women as well as presenting a risk to immunocompromised individuals [8,9,10,11,12,13]. More recently, *C. abortus* has been associated with atypical pneumonia in a laboratory worker following exposure to the organism in a research facility [14].

Chlamydial abortion is a consequence of the organism’s efficiency in colonizing the placenta, initially via the trophoblast, and inducing pathological changes that compromise the developing fetus. Infection spreads rapidly, leading to vasculitis and necrosis of the cotyledonary and intercotyledonary membranes with the typical associated macroscopic lesions and exudate [15], culminating in either late-term abortion, stillbirths, the birth of weak lambs, or potentially a combination of these outcomes in multiparous ewes [1,16]. OEA spreads to naïve animals via oropharyngeal exposure, possibly in the tonsils [17], most likely at lambing or abortion, when infected ewes shed large amounts of *C. abortus* in vaginal discharges and infected placentas [3]. A state of latency ensues in non-pregnant animals until the onset of pregnancy, when the pathogen recrudesces and rapidly multiplies, leading to pregnancy failure [15]. Ewes that have experienced disease will develop protective immunity to subsequent abortions but may excrete infectious organisms in the next lambing season, thereby posing a risk to naïve animals [18].

The most effective method of preventing infection and abortion in flocks is through vaccination. Several approaches have been taken in the effort to develop vaccines to protect against *C. abortus* infections, including inactivated whole organisms [19,20,21], live attenuated organisms [22,23], subcellular preparations [24], and recombinant subunit antigens [25,26,27], with varying levels of success. A whole-organism formalin-inactivated vaccine developed in the UK was successfully deployed for many years [28] until vaccine breakdown and reduced efficacy was observed [29], resulting in its subsequent withdrawal from the market. This led to the development of a live attenuated vaccine based on the 1B strain of *C. abortus* [22,30], resulting in the two commercial vaccines that are currently available in the UK and throughout Europe (Enzovax^®^, MSD Animal Health UK Limited, Milton Keynes, UK, and Cevac^®^ Chlamydia Ceva Animal Health Ltd., Wooburn Green, UK), both of which are delivered as a single inoculation. There is also an inactivated combined vaccine against *Chlamydia abortus* and *Salmonella Abortusovis* available in the UK called INMEVA^®^ (HIPRA UK & Ireland Ltd., Nottingham, UK), but it requires two doses and a booster within a year [31]. Although the live attenuated vaccines generally confer good protection, PCR-RFLP discriminatory analysis of placental samples and isolation of *C. abortus* from cases of abortion in vaccinated flocks have identified the 1B vaccine strain as the causative agent, thus highlighting problems and safety concerns [32]. Additionally, sequencing and comparative genomic analyses have brought into question the attenuated nature of this vaccine strain and, hence, its potential to cause disease [33]. Furthermore, recent manufacturing problems have resulted in a shortage of the vaccine in the UK, thus impacting the sheep industry and effective disease control [34]. Consequently, the safety issues surrounding the live vaccines have led us to develop a safer, more stable, and efficacious subcellular vaccine for the protection of ewes against OEA. The antigenic component, based on the chlamydial outer membrane complex (COMC) and previously having been shown to be protective [24,35], comprises the protective and strongly antigenic major outer membrane protein (MOMP) [36].

Previously, we have shown, through a series of experimental trials in pregnant sheep [36,37,38], that the *C. abortus* COMC vaccine can be delivered in a single dose of as little as 7 µg antigen when formulated with the adjuvant Montanide ISA 70VG [39] and still remain efficacious. In this study, we have compared the effect of replacing this adjuvant with either Montanide ISA 61 VG or QuilA on protective efficacy, using a suboptimal dose of COMC antigen (2.5 µg) to allow for the detection of any improvements in efficacy. Vaccine efficacy was evaluated as in our previous vaccine trials by assessing reductions in the number of abortions, in placental pathology, and in the number of organisms shed at parturition compared to an unvaccinated challenge control group, utilizing our well-established pregnant sheep challenge model [24,37,38,40,41].

## 2. Materials and Methods

### 2.1. Ethics Statement

The entire experimental protocol was conducted in strict accordance with the Animals (Scientific Procedures) Act 1986 and complied with both UK Home Office Inspectorate regulations and ARRIVE guidelines 2.0 [42]. The animal study was approved by the Moredun Experiments and Ethical Review Committee (Permit number: E30/17) on 15 August 2017. Safety and responses to vaccination and challenge, as well as monitoring of the animals throughout the study and appropriate veterinary care, were assessed and carried out as detailed in our previous connected studies [36,37,38].

### 2.2. Preparation of C. abortus COMC Antigen and Formulation of Vaccines

Elementary bodies (EBs) were isolated and purified from *C. abortus* (strain S26/3) infected McCoy cells [43] and used to prepare the COMC vaccine antigen, as described previously [36,37,38,44]. COMC antigen was quantified for formulation into the vaccines and for use in the cellular immunological assays, as previously described [24,36,37,38].

Four different COMC vaccine preparations were formulated. Group 1 and 2 vaccines were adjuvanted with Montanide™ ISA 70 VG (Seppic SA, La Garenne-Colombes, France), using a ratio of adjuvant (oil phase)/antigen (aqueous phase) of 70/30 (weight/weight), providing formulations containing final concentrations of 20 µg and 2.5 µg equivalent MOMP protein per 1 mL dose, respectively. The group 3 vaccine was formulated with Montanide™ ISA 61 VG (Seppic SA), using a ratio of adjuvant (oil phase)/antigen (aqueous phase) of 60/40 (weight/weight), providing a formulation containing 2.5 µg equivalent MOMP protein per 1 mL. All three vaccines were emulsified using an IKA T 25 Ultra-Turrax^®^ T25 homogenizer (Scientific Laboratories Supplies Ltd., Newhouse, UK; #HOM2000) at a high shear rate at room temperature, according to the manufacturer’s protocol. The group 4 vaccine was adjuvanted with QuilA, provided by parasitology colleagues at Moredun [45], by mixing the adjuvant and antigen together to give a final concentration of 0.5 mg saponin and 2.5 µg equivalent MOMP protein per 1 mL dose. Vaccines were prepared and stored at 4 °C for one month prior to administration to ensure stability.

### 2.3. Preparation of C. abortus S26/3 Challenge Inoculum

*C. abortus* strain S26/3 was cultivated in the yolk sacs of specific pathogen-free hens’ eggs using standard techniques [46], pooled and suspended in PBS, and stored in liquid nitrogen until required. The number of inclusion-forming units (IFUs) present in the challenge material was determined via titration in McCoy cell monolayers grown on coverslips, as previously described [36,37]. The challenge inoculum was removed from liquid nitrogen storage on the day of use and diluted in PBS to provide 10^6^ IFUs of *C. abortus* per mL.

### 2.4. Experimental Design

A total of 225 primiparous 1–2-year-old crossbreed Scotch Mule ewes were sourced from disease-free EAE-accredited flocks participating in the Premium Sheep and Goat Health Schemes (run by Scotland’s Rural College Veterinary Services [47]). All animals were pre-screened, as previously described, using a rOMP90-3 enzyme-linked immunosorbent assay (ELISA) [48] and an in vitro lymphocyte stimulation assay [41]. Sheep with pre-existing antibodies to OMP90-3 or producing IFN-gamma (IFN-γ) responses greater than 200 pg/mL (approximately more than two standard deviations from the medium controls) were excluded from the study. Sheep were additionally pre-screened for bovine viral diarrhea virus and border disease via the Moredun Virus Surveillance Unit. Selected sheep were randomly allocated to six groups (groups 1–5 each contained 39 animals, and group 6 contained 7 animals). Eight weeks before mating, group 1–4 animals were vaccinated with a 1 mL vaccine dose administered intramuscularly (i.m.) using a 19G 1” needle on the left side of the neck as follows: group 1, 20 µg MOMP/Montanide ISA 70 VG; group 2, 2.5 µg MOMP/Montanide ISA 70 VG; group 3, 2.5 µg MOMP/Montanide ISA 61 VG; and group 4, 2.5 µg MOMP/QuilA. Group 5 (challenge controls) and 6 (negative controls) animals were not vaccinated. Six weeks following vaccination, all ewes were synchronized using Chronogest^®^ CR 20 mg controlled-release vaginal sponges (MSD Animal Health UK Ltd., Milton Keynes, UK) over two weeks and then mated. All pregnant vaccinated ewes (groups 1–4) and group 5 challenge control ewes were inoculated subcutaneously (s.c.), using a 19G 1” needle, over the left prefemoral lymph node with 2 mL of challenge inoculum containing 2 × 10^6^ IFU of *C. abortus* S26/3 at day 70 of gestation. Group 6 animals served as non-vaccinated and non-challenged negative controls and were placed in separate housing remote from the challenged animals. All animals were fed on a supplemented diet appropriate for pregnant ewes, and hay and water were offered ad libitum. The clinical outcome of each ewe was recorded, along with the weight and sex of each lamb/fetus immediately after delivery. A ewe was considered to have aborted if it delivered at least one dead lamb, or a weak lamb that had to be euthanized on animal welfare grounds or died within 48 h of birth, and when chlamydial EBs/DNA could be demonstrated in the fetus, placenta, or uterine discharge using stained smears, quantitative real-time polymerase chain reaction (qPCR), or through pathological investigation. Animals subsequently found not to be pregnant after the expected parturition date were withdrawn from the study.

### 2.5. Sample Collection and Processing

**Figure 1:**
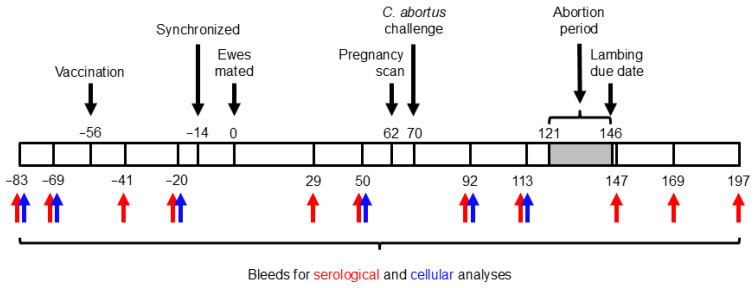
Experimental schedule. The numbers above and below the bar indicate days prior to (negative numbers) or after (positive numbers) mating.

The study design and timeline are illustrated in Figure 1. Blood samples were taken from all ewes prior to vaccination and every 2–4 weeks throughout the study. Blood (10 mL) was collected via jugular venipuncture into BD Vacutainer^®^ serum tubes (Fisher Scientific, Loughborough, UK; #12957686) for serological analysis via an ELISA, while an additional 20 mL was collected into BD Vacutainer^®^ heparin tubes (Fisher Scientific; #13171543) for cellular analyses (see Section 2.9). Following abortion or the delivery of lambs, placentas were collected into individual bags labeled with the ewe number and transported to the post-mortem room, where macroscopic evaluation was carried out by estimating the percentage of surface area affected by typical OEA lesions, as previously described [36]. All placentas were sampled by excising two affected cotyledons. If no affected cotyledons were evident, then apparently unaffected cotyledons were randomly selected. One half of every selected cotyledon was placed in a sterile 7 mL container, from which impression smears were made and subsequently stained with a modified Ziehl–Neelsen (mZN) [1] stain for detecting chlamydial organisms and for qPCR [49]. The other half of the cotyledons were fixed in a pre-filled 10% neutral-buffered formalin container (CellPath™ Cellstor Pot; Fisher Scientific; #13191184) for conventional histopathological examination and labeling of chlamydial antigen. To ensure sampling consistency and immediately following expulsion of the placenta, vaginal swabs (Technical Service Consultants™ Hygiene Swab; Fisher Scientific; #12749945) were taken from each animal for qPCR to measure the bacterial load [49], as well as for mZN when the placenta could not be retrieved. For any suspected non-chlamydial causes of fetal abortion, brain, lung, heart, and liver tissues were collected in 10% BF for histopathological investigation and IHC. This allowed us to discount other common abortifacient bacterial pathogens as a cause of death, as well as possible suffocation resulting from dystocia that can occur during lambing [37].

### 2.6. Chlamydia abortus Quantitative Real-Time PCR

Two vaginal swabs, collected from each animal following delivery of the placentas, were processed using a DNeasy^®^ Blood and Tissue Kit (Qiagen Ltd., Crawley, UK; #69504) and extracted DNA analyzed via real-time PCR (qPCR), based on the *C. abortus* OmpA gene (CAB048, accession number CR848038), as previously described [36,37,49]. Samples were tested in triplicate and quantified against a standard curve prepared from *C. abortus* genomic DNA, with the results expressed as the number of genome copies per 1 µL of swab DNA. A cut-off of 100 copies was applied based on the qPCR data obtained from negative control animals in previous pathogenesis studies conducted over the last 15 years, as detailed previously [36].

### 2.7. Histopathological and Immunohistochemical Analyses of Tissues

Fixed (in 10% BF) placental and fetal tissues were processed, embedded in paraffin wax and sectioned, as previously described [37,50]. Then, 5 µm serial sections of embedded tissues were stained with hematoxylin and eosin for histopathological examination or were labeled for IHC with an anti-chlamydial lipopolysaccharide (LPS) monoclonal antibody (mAb 13/4; Santa Cruz Biotechnology, Inc., Heidelberg, Germany; #sc-101593), as previously described. A goat anti-mouse IgG conjugate (Dako EnVision™+ System HRP-labeled polymer (mouse); Agilent Technologies Denmark ApS, Glostrup, Denmark; #K4001) was used to detect and visualize bound mAb 13/4. Sections were counterstained with hematoxylin and mounted (Histomount^TM^, Thermo Fisher Scientific; #008030). A positive control section of placental tissue from a known ovine chlamydial abortion case and negative control sections were routinely included in every run [50].

### 2.8. Immunological Analyses

Circulating antibodies to *C. abortus* were detected in serum recovered from blood samples taken throughout the course of the study and analyzed using the indirect rOMP90B-3 ELISA, as previously described [48]. Results were normalized using positive (from convalescent *C. abortus* infected sheep) and negative control sera and expressed as a percentage of the positive control [(OD sample − OD negative control)/(OD positive control − OD negative control)] × 100, as previously described [48].

Heparinized whole blood collected from animals was used to prepare peripheral blood mononuclear cells (PBMCs), which were counted and adjusted to 2 × 10^6^ cells/mL in complete Iscove’s Modified Dulbecco’s Medium (IMDM). Cells were cultured in 96-well sterile U-bottom plates (Thermo Fisher Scientific (Nunc^TM^); #168136) for 96 h, as previously described [37,41]. Then, 100 μL each of purified *C. abortus* COMC antigen (0.5 μg/mL), purified and UV-inactivated *C. abortus* EB antigen (1 µg/mL) [41], ConA (5 μg/mL; concanavalin A from *Canavalia ensiformis*, Merck Life Science UK Ltd. (Sigma-Aldrich Co.), Gillingham, UK; #C0412), and medium alone were added to cells in quadruplicate wells for each animal and time point (see Figure 1). Antigen-specific recall responses and assay stimulation controls were assessed by analyzing the collected culture supernatants for the production of pro-inflammatory (IFN-γ and interleukin (IL)-17A) and anti-inflammatory/regulatory (IL-4 and IL-10) cytokines, as previously described [41]. The pro-inflammatory IL-17A was measured via a specific sandwich ELISA using our generic cytokine ELISA protocol [41], using the capture monoclonal antibody clone MT49A7 at 1 µg/mL, detection mAb clone MT51B8-biotin at 0.5 µg/mL, and an extended recombinant standard range from 2000 to 10 pg/mL (ELISA Flex: Sheep IL-17A (HRP), Mabtech AB, Nacka Strand, Sweden; #3127-1h-20).

### 2.9. Statistical Analyses

Abortion rates across treatment groups (groups 1–5) were analyzed using a standard binomial generalized linear model (GLM) with a logit link function, fitted via maximum likelihood estimation. For the overall comparison between the challenge control group (group 5) and the vaccinated groups (groups 1–4), data from the vaccinated groups were pooled into a single treatment group, aggregating the numbers of animals that lambed or aborted.

Bacterial loads quantified via qPCR were compared among treatment groups using non-parametric contrast tests based on global rankings [51]. Specifically, Dunnett’s contrasts were applied to compare vaccinated groups (groups 1–4) against challenge control (group 5), while Tukey’s contrasts were applied to assess the differences among vaccinated groups themselves. Similarly to abortion rates, gross infection rates were analyzed using a binomial GLM, with group comparisons based on estimated marginal means.

Longitudinal observations of serological and cytokine responses were modeled using ordinary linear mixed models (LMMs), fitted by restricted maximum likelihood to rank-based inverse normal transformed data (Blom’s transformation). The treatment group, bleed, and the interaction between them were included as fixed effects, whereas the animal ID was used as a random effect. A similar LMM approach was employed to compare responses between lambed and aborted animals. Post hoc comparisons were performed using estimated marginal means.

All statistical analyses were conducted using R version 4.5 [52]. Where applicable, *p*-values derived from multiple statistical testing were adjusted for false discovery rate using the Benjamini–Hochberg’s procedure [53]. Statistical significance was concluded at the ordinary 5% level.

## 3. Results

### 3.1. Clinical Outcome

The pregnancy outcomes for each of the experimental and control groups are summarized in Table 1 and given in full detail for each individual animal in Table S1. No adverse responses were observed in any of the animals to either vaccination or to challenge, which is consistent with what we have observed in our previous studies [37,38,48]. As expected, no abortions occurred in any of the negative control sheep (group 6), with all lambs being delivered healthy and close to their expected parturition dates. The gestational average of this group was 146.9 days (range of 144–149 days; Table S1), which is in keeping with an average length of gestation of 147 days and range of 142–152 days for sheep in general. Most of the ewes (129 of 143) in the vaccinated groups (groups 1–4 in Table 1) delivered healthy live lambs with a mean length of gestation of 146.2 days (range of 138–151 days; Table S1), which is very similar to that observed in the negative control group and similar to that observed in the challenge control group, which had an average of 144.7 days (range of 138–150; Table S1). Overall, there was little difference in the average length of gestation of the animals that lambed in the vaccinated and negative control groups, with that of the challenge control group being slightly lower. Consistent with previous studies, the average length of gestation was lower by 6–10 days for the animals that aborted in the vaccinated and challenge control groups.

Single abortion events occurred in groups 1 (one set of triplets; Table S1) and 2 (one single lamb), receiving the same adjuvant (ISA 70 VG) but very different doses (20 versus 2.5 μg). Slightly more abortions (n = 3) occurred in group 3 (two sets of twins and one individual) which received a different adjuvant, namely ISA 61 VG, and even more in group 4 (n = 9; four sets of twins and five individuals), which received the QuilA adjuvant. The greatest number of abortions occurred in the challenge control group (n = 14; seven sets of twins and seven individuals) and resulted in the highest abortion rate (36.8% versus 2.7–23.7%). Although there were differences in the number of abortions occurring between the different vaccination groups (groups 1–4), the abortion rate across the four vaccinated groups overall (9.8%; 14 of 143 animals) was statistically significantly reduced compared to the challenge control group 5 (*p* = 0.0001). Individually, no statistically significant reduction in abortion rate was obtained for group 4 (*p* = 0.2147). When comparing the effectiveness of the three 2.5 μg vaccine formulations (groups 2–4) with the benchmark 20 μg formulation (group 1), we observed no statistically significant difference in the number of abortions occurring in groups 2 and 3 compared to group 1 (*p* > 0.3270), but there was a statistically significant increase in the number of abortions occurring in group 4 (*p* = 0.0259).

A further three lambs were found dead in the vaccinated groups 1 and 2, one of which was a tiny, mummified fetus delivered alongside a normal lamb at the expected parturition date (day 146), but none of these showed any bacteriological or pathological evidence of OEA (Table 1 and Table S1).

### 3.2. Estimation and Detection of C. abortus Infection and Pathogen Load

Following parturition, whether resulting in a normal lambing or abortion, a total of 297 of 309 placentas were successfully recovered and examined for evidence of gross pathology that is typically associated with OEA. Gross pathology was much more evident and extensive for the placentas associated with the aborted lambs from the vaccinated/challenged and challenge control ewes than for those that successfully lambed (Table 2 and Table S1). Generally, the lesions covered most of the surface of the placentas retrieved from ewes that aborted (n = 23; range of 60–100%), although there were exceptions where the coverage was much lower (n = 13; range of 1–50%). No gross pathology was observed on 198 placentas associated with vaccinated/challenged and challenge control ewes only producing live lambs, with a further 42 placentas exhibiting varying degrees of lesion coverage (range of 1–80%). Amongst those exhibiting pathology in these 42 placentas, we did note that the majority of these occurred in the group 4 QuilA vaccinated (n = 17) and challenge control (n = 19) ewes.

The mZN placental smear results were very similar to those obtained for gross pathology and, in fact, were identical for groups 1 and 2, with organisms detected in a few extra placentas from the lambed ewes of vaccinated groups 3 and 4 (extra 4 and 2, respectively) and the challenge control group (extra 2) (Table 2 and Table S1). Smear results for the placentas from aborted animals perfectly matched the gross pathology results. However, one of two placentas from an animal in group 5 that aborted (ewe 09944 in Table S1; produced one live and one aborted lamb) was not retrieved for the aborted lamb, and the placenta from the live lamb was negative in terms of gross pathology and mZN placental smear (Table 2), but this ewe was positive via qPCR of the post-partum vaginal swab.

Overall, qPCR analysis of swabs of post-partum vaginal fluids taken following delivery of afterbirths added a further and large increase in the sensitivity of pathogen detection (herein referred to as ‘bacterial load’) and, hence, the number of lambed animals that were deemed bacteriologically positive (Table 2 and Table S1). This was particularly evident in the vaccinated groups 1 to 3, where an additional 12 to 16 animals were deemed positive, leading to positivity rates of 40–67%, although it was clear that the bacterial loads were overall still very low in the these groups, as reflected by the low geometric means (107–240 *C. abortus* genome copies per µL of extracted material; Table 2). Although these geometric means were very low, they were higher than observed in our negative control animals (13 genome copies), which largely resulted from one ewe in group 1 having a bacterial load (1.95 × 10^5^ genome copies) comparable to what is often observed in aborted animals, while there were three (2.0 × 10^4^–2.68 × 10^6^ genome copies) and four (1.02 × 10^3^–3.96 × 10^5^ genome copies) such ewes in groups 2 and 3, respectively (Table S1). In contrast to these three groups, the group 4 vaccinated lambed ewes had a much higher geometric mean (41,402 genome copies), reflecting a much higher proportion of bacteriologically-positive ewes (93% positive), most of which had very high bacterial loads (1.18 × 10^3^–5.52 × 10^6^ genome copies), and very comparable to what we observed in the challenge control group (95.8% positive; 1.11 × 10^3^–5.35 × 10^6^ genome copies) (Table 1 and Table S1). As indicated, the geometric means of the aborted animals across all vaccinated and challenge control groups were very high (1.07 × 10^6^–3.21 × 10^6^ genome copies; Table 2) and reflective of the observed individual high bacterial loads (5.26 × 10^4^–1.41 × 10^7^ genome copies; Table S1).

Overall, there was a statistically significant reduction in the bacterial load in vaccinated groups 1 to 3 (*p* < 0.0001) but not in group 4 (*p* = 0.5509) when compared to challenge control group 5. There was no statistically significant difference in the bacterial loads of groups 2 and 3 when compared to the benchmark group 1 (*p* > 0.3177), but group 4 had a statistically significant higher bacterial load (*p* < 0.0001). Similarly, when taking into consideration all of the bacteriological data, including the gross pathology, the presence of organisms in placental smears, and bacterial loads on post-partum vaginal swabs (herein referred to as the ‘infection rate’), the infection rates were statistically significantly higher for the challenge control group and vaccinated group 4 animals when compared to the benchmark group 1 (*p* < 0.0009), with the data not showing any statistically significant distinction between groups 1 to 3 (*p* > 0.0390).

### 3.3. Histology and Immunohistochemical Analysis

Placentas and fetuses were randomly selected from each group for both histological and IHC analyses. All analyzed samples from all abortion cases revealed pathology that is associated with OEA [40,50,54], specifically a suppurative necrotizing placentitis with vasculitis and positive labeling for *C. abortus* antigen via IHC. There were no observable differences in the pathology from the placental and fetal tissues from the animals that aborted in the vaccinated groups and the challenge control group. In the case of the three lambs that were found dead from groups 1 and 2 (see Table 1 and Table S1), histological and IHC investigations did not reveal any OEA lesions or any positive *C. abortus* labeling in any of the examined tissue samples, and no other pathological cause was able to be determined.

### 3.4. Serological Responses

**Figure 2:**
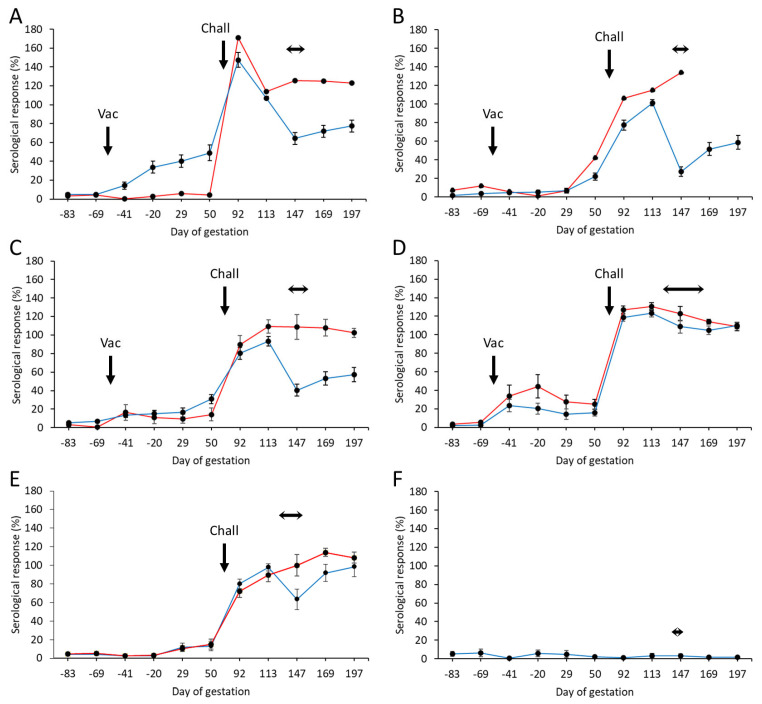
Serological responses following vaccination with different COMC/adjuvant formulations and *C. abortus* challenge. Detection of *C. abortus* antibody in ewes vaccinated (56 days prior to mating) with a single dose (Vac) of either 20 μg (**A**) or 2.5 μg (**B**–**D**) COMC antigen formulated with Montanide ISA 70 VG (**A**,**B**), Montanide ISA 61 VG (**C**), or QuilA (**D**) and challenged (Chall) on day 70 of gestation with *C. abortus* strain S26/3. Unvaccinated challenged (**E**) and unvaccinated non-challenged (**F**) ewes were included as positive and negative control groups. Data are separated into lambed (blue lines) versus aborted (red lines). Data points represent the arithmetic mean values for each cellular bleed, and error bars represent the standard error of that mean (SEM). Here, 100% is equivalent to an OD450 nm of 2.25. The lambing/abortion period for each group is indicated by the horizontal double-headed arrows. Note that the panel B aborted animal is missing the last two bleeds, as she had to be euthanized at parturition on welfare grounds.

From the 225 pre-screened ewes, a total of 202 were selected as negative for *C. abortus* before commencing the study and allocated to the six groups, as detailed in Section 2.5. The mean specific antibody responses for each of the experimental and control groups throughout the study, separated into those that aborted and those that lambed, are shown in Figure 2, while individual animal responses are detailed in Table S2. Following vaccination, antibody responses were elevated in three of the four (groups 1, 3, and 4) vaccinated groups. Very little response was detectable in group 2 (Figure 2B). The response in group 1 (Figure 2A) was statistically significantly higher than in the other groups and more sustained prior to challenge (*p* = 0.0018), while it was more transient in nature in group 4 (Figure 2D), slowly declining in titer until challenge. In general, there was very little antibody response in the vaccinated aborted animals, the exceptions being the group 2 animal, where there was a delayed elevated response just prior to challenge, and the animals in group 4, where the mean response was statistically greater in the aborted than the lambed animals (*p* = 0.0305).

Following challenge, antibody responses rapidly increased in all vaccinated (Figure 2A–D) and challenge control (Figure 2E) groups. The responses were statistically significantly higher in the first bleed at three weeks post-challenge for the group 1 animals (Figure 2A) compared to the other vaccinated groups (*p* < 0.0276) and the challenge control group (*p* < 0.0001). At the time of parturition and following that period, there was a noticeable difference in responses between lambed and aborted animals, where those that aborted generally had a statistically higher antibody response than those that lambed (*p* < 0.0001). We noted a rapid drop in antibody responses at parturition in lambed animals in the vaccinated and challenge control groups, which then started to increase post-parturition. The exception to this was for group 4 vaccinated animals, where both aborted and lambed animals had very similar antibody profiles, which gradually decreased following parturition.

All negative control group animals remained serologically negative throughout the study (Figure 2F).

### 3.5. Cellular Responses

The cohort of 225 sheep was pre-screened for cellular recall responses to *C. abortus* antigens (COMC vaccine antigen and EBs) and the T-cell mitogen ConA on two occasions prior to vaccination (Figure 1). As part of the selection of the final cohort, animals with high IFN-γ responses to the medium alone and *C. abortus* antigens and/or poor responses to ConA were excluded. The final 202 animals selected were also confirmed as serologically negative, as described in Section 3.4. The identified sheep were randomly split into groups assigned on the basis of similar proportions of animals with a range of lower, medium, and higher IFN-γ responses to ConA, as we have undertaken for previous studies [41].

**Figure 3:**
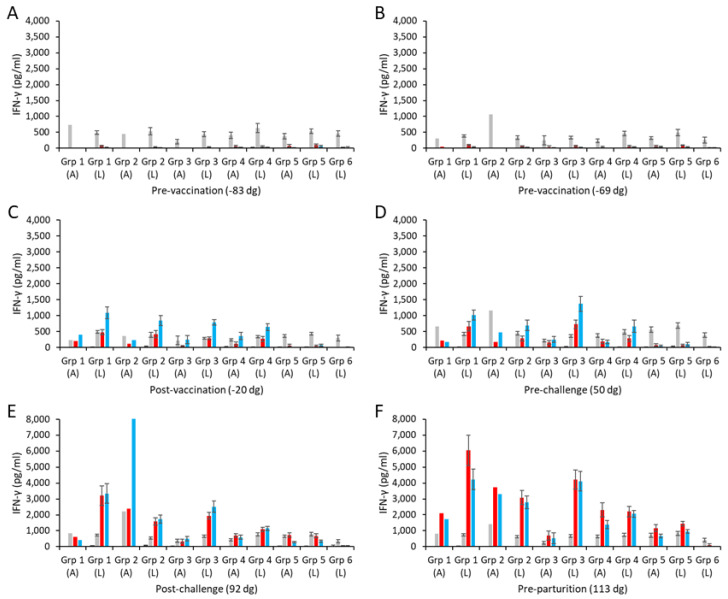
Interferon-γ responses following vaccination with different COMC/adjuvant formulations and *C. abortus* challenge. Peripheral blood mononuclear cells (PBMCs) were purified from whole blood (as described in Section 2) for the vaccinated, challenge control, and negative control groups and were collected at the indicated time points (day of gestation; dg) prior to and post-mating (also see Figure 1). Bleeds were taken pre-vaccination (**A**,**B**), post-vaccination (**C**), pre-challenge (**D**), post-challenge (**E**), and pre-parturition (**F**). Antigen-specific IFN-γ recall responses were assessed via analysis of culture supernatants resulting from the stimulation of purified PBMCs in vitro, using medium only as an unstimulated cell control (black bars; very low relative to mitogen and antigens) and Concanavalin A (ConA) as a positive control (grey bars). UV-inactivated *C. abortus* EB (red bars) and COMC (vaccine antigen; blue bars) antigens were used for measuring chlamydial antigen-specific stimulation. Data have been separated into lambed and aborted for each group, and values represent the means for each cellular bleed, while error bars represent the standard error of that mean (SEM). Note the different scales for the ordinate axis in panels E and F.

Specific mean cellular IFN-γ responses for the aborted and lambed animals in each of the vaccinated challenged groups and control groups are shown in Figure 3 (raw data are shown in Table S2). Overall, the responses to the medium alone and to the positive control mitogen ConA were broadly consistent (Figure 3A–F), while the chlamydial antigen responses of the unvaccinated, unchallenged group 6 sheep remained negligible throughout the study (Figure 3A–F).

The IFN-γ responses to UV-inactivated EB antigen and COMC in the two pre-vaccination bleeds (Figure 3A,B) were mostly negligible across all the vaccinated groups and similar to those with medium alone. Following immunization, COMC vaccine antigen-specific IFN-γ responses were greatly elevated across all the vaccinated groups (groups 1–4; Figure 3C) at levels statistically greater than observed to chlamydial EBs and ConA (*p* < 0.0001). A similar pattern as this was also observed pre-challenge (Figure 3D), while post-challenge (Figure 3E), the COMC and EB responses appeared not statistically distinguishable from each other (*p* = 0.1773), with the notable exception of the aborted animal in group 2.

Comparison of the specific COMC IFN-γ responses between the ISA 70 VG-adjuvanted groups (groups 1 and 2) revealed that the group receiving the higher vaccine antigen dose (group 1) had statistically stronger COMC responses over the study duration (Figure 3C–F) (*p* = 0.0041), with the notable exception of the single aborted animal bleed in group 2 post-challenge (Figure 3E). The vaccine groups (with ISA 70 VG, 61 VG, and QuilA adjuvants) with the 2.5 µg/mL vaccine antigen dose had statistically greater COMC responses than the challenge-only control from the post-vaccination bleed (Figure 3C–F, *p* < 0.0001). Comparing the three 2.5 µg/mL vaccine antigen dose groups with each other, we noted that while they led to IFN-γ mean responses that were statistically different from each other (*p* < 0.0330), group 4 exhibited the lowest, followed by groups 2 and 3 in increasing order. Analysis of the post-challenge bleeds revealed elevated COMC IFN-γ responses in all challenged groups (groups 1–5, Figure 3E). Overall, the pre-parturition bleeds had the greatest vaccine-specific IFN-γ responses (Figure 3F), where responses in the vaccinated challenged groups were statistically stronger than the challenge-only group (*p* < 0.0001). Interestingly, when analyzing all COMC vaccine groups by outcome across the same bleeds, we observed that lambed animals mostly had higher responses than the aborted animals (Figure 3C–F).

**Figure 4:**
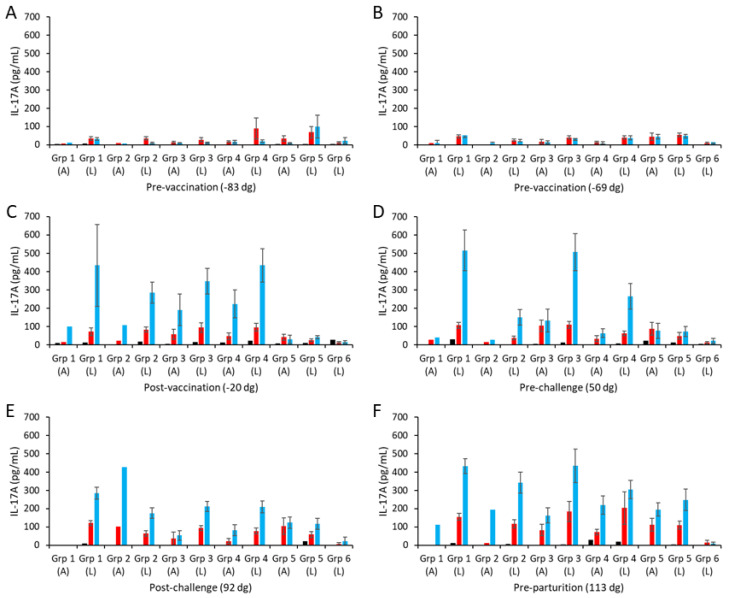
Interleukin-17A responses following vaccination with different COMC/adjuvant formulations and *C. abortus* challenge. Peripheral blood mononuclear cells (PBMCs) were purified from whole blood (as described in Section 2) for the vaccinated, challenge control, and negative control groups and were collected at the indicated time points (day of gestation; dg) prior to and post-mating (also see Figure 1). Bleeds were taken pre-vaccination (**A**,**B**), post-vaccination (**C**), pre-challenge (**D**), post-challenge (**E**), and pre-parturition (**F**). Antigen-specific IL-17A recall responses were assessed via analysis of culture supernatants resulting from the stimulation of purified PBMC in vitro, using medium only as an unstimulated cell control (black bars; very low relative to mitogen and antigen). UV-inactivated *C. abortus* EB (red bars) and COMC (vaccine antigen; blue bars) antigens were used for measuring chlamydial antigen-specific stimulation. Note that ConA data have not been included so that the EB and COMC responses can be more easily observed (see Table S2 for ConA data). Data have been separated into lambed and aborted for each group, and values represent the means for each cellular bleed, while error bars represent the standard error of that mean (SEM).

PBMCs from all groups consistently produced IL-17A in response to ConA stimulation over time (Table S2) that was generally several orders of magnitude higher than the ConA-mediated IFN-γ responses. Consistently low IL-17A responses to the chlamydial antigens were observed pre-vaccination, with some inter-sample point variability at the group level (Figure 4A,B; note that ConA responses are presented in Table S2). Following vaccination and challenge, animals with a lambed outcome had the highest COMC IL-17A responses, which were not statistically significant from animals that aborted (Figure 4C–F; *p* = 0.4589) but were statistically significantly greater than for the non-vaccinated controls (Figure 4C–F; *p* = 0.0010). COMC stimulated statistically stronger IL-17A responses than UV-inactivated EBs post-challenge, indicative of vaccine-induced immune priming (Figure 4E,F; *p* = 0.0038).

The higher COMC vaccine antigen dose adjuvanted with Montanide ISA 70 VG (group 1) stimulated higher IL-17A responses, albeit not statistically significantly higher, than for the lower dose group (group 2; Figure 4C–F, *p* = 0.1297), as observed for IFN-γ. Similarly, comparing the three 2.5 µg/mL vaccine antigen dose groups with each other, we observed no statistically significant differences between them (*p* > 0.1354), although group 3 had a higher response, which was not distinguishable from that of group 1 pre-challenge (Figure 4D). Overall, only vaccine groups 1 and 3 had statistically significantly higher IL-17A responses than observed for the challenge control group (*p* = 0.0104).

**Figure 5:**
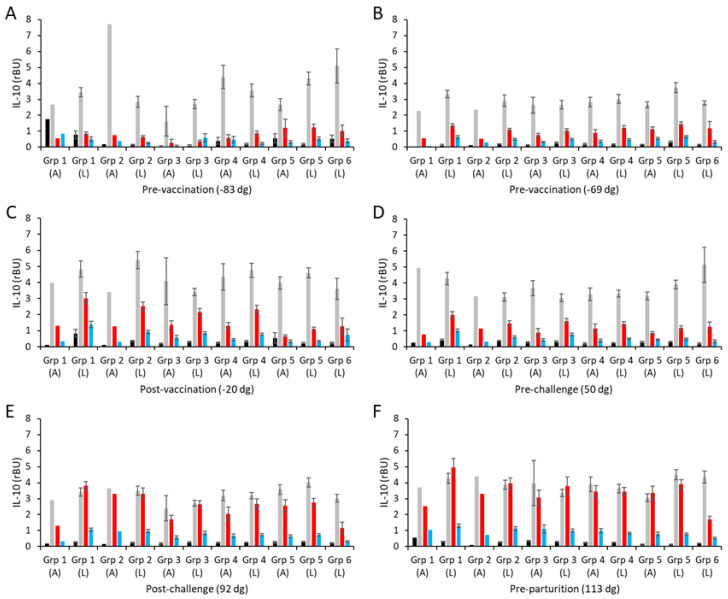
Interleukin-10 responses following vaccination with different COMC/adjuvant formulations and *C. abortus* challenge. Peripheral blood mononuclear cells (PBMCs) were purified from whole blood (as described in Section 2) for the vaccinated, challenge control, and negative control groups and were collected at the indicated time points (day of gestation; dg) prior to and post-mating (also see Figure 1). Bleeds were taken pre-vaccination (**A**,**B**), post-vaccination (**C**), pre-challenge (**D**), post-challenge (**E**), and pre-parturition (**F**). Antigen-specific IL-10 recall responses were assessed via analysis of culture supernatants resulting from the stimulation of purified PBMCs in vitro, using medium only as an unstimulated cell control (black bars; very low relative to mitogen and antigen) and Concanavalin A (ConA) as a positive control (grey bars). UV-inactivated *C. abortus* EB (red bars) and COMC (vaccine antigen; blue bars) antigens were used for measuring chlamydial antigen-specific stimulation. Data have been separated into lambed and aborted for each group, and values represent the means for each cellular bleed, while error bars represent the standard error of that mean (SEM).

The culture supernatants from the in vitro PBMC recall assays were also screened for anti-inflammatory IL-10 responses. The resulting mean IL-10 responses are shown separately for the aborted and lambed animals for each group in Figure 5 (raw data are shown in Table S2). In both pre-vaccination bleeds (Figure 5A,B), IL-10 was detected in PBMCs stimulated with ConA, while the levels detected in cultures with both chlamydial antigens or medium alone were low and at the limits of sensitivity of the ELISA. The intra-group IL-10 responses were consistently between 3–5 rBU/mL to ConA (Figure 5A,B; Table S2).

Generally, the vaccine antigen COMC, ConA, and medium-alone IL-10 responses across vaccinated and control groups were consistent across all sampling time points, except for a few occasional cases, showing that there was little IL-10 response to vaccination (groups 1–4; Figure 5A–F) or experimental challenge (group 5; Figure 5A–F). In contrast to IFN-γ and IL-17A, the IL-10 responses to UV-inactivated EBs are higher than to the COMC but not statistically significantly different to the non-vaccinated, non-challenged controls of group 6 (Figure 5A–D, *p* < 0.0001). Post-challenge (Figure 5E) and pre-parturition (Figure 5F), the IL-10 levels in response to UV-inactivated EBs were elevated in the vaccinated and challenge-only groups (groups 1–5, *p* < 0.0001) and statistically significantly higher than the responses observed in the unvaccinated, unchallenged negative control group 6 (Figure 5E,F, *p* < 0.0001). In slight contrast to these IL-10 production data, there was no evidence of any counter-regulatory driven IL-4 production following either vaccination or experimental challenge, with both COMC and UV-inactivated EB responses being consistently flat throughout (Table S2). Therefore, overall, while the different vaccine formulations have primed for pro-inflammatory cytokine production post-challenge to varying degrees, they have not primed for regulatory/anti-inflammatory IL-10 and IL-4 production.

## 4. Discussion

In previous studies, we have identified a subcellular detergent-extracted outer membrane preparation of *C. abortus* (COMC or chlamydial outer membrane complex) as being highly protective in our pregnant sheep model for OEA [38], as well as demonstrating that this experimental vaccine can be administered in a single inoculation of 7–20 µg antigen without compromising efficacy [36,37]. The COMC antigen in this experimental vaccine was adjuvanted with the Seppic Montanide series adjuvant ISA 70 VG, which the manufacturer had recommended we used to target both humoral and cellular responses in sheep. Subsequently a publication on the immunogenicity of a *Mycobacterium avium* subsp. *paratuberculosis* vaccine suggested that ISA 70 VG was much less effective at eliciting IFN-γ responses compared to a newer adjuvant, ISA 61 VG [55]. This resulted in the manufacturer recommending ISA 61 VG over ISA 70 VG for use in small ruminants. Despite our studies with our COMC vaccine in the last three trials finding that ISA 70 VG was effective in driving strong chlamydial antigen-specific IFN-γ responses [36,37,38], we nevertheless thought it was important to compare the two adjuvants in this study. QuilA adjuvant and other saponins, on the other hand, have been widely used in a variety of veterinary vaccines, activating both humoral and the cell-mediated immune responses to a broad range of bacterial, viral, and parasitic antigens [56,57,58], including against OEA [59]. Furthermore, colleagues at Moredun have had great success with using QuilA in their experimental helminth and ectoparasite vaccines [45,60], hence the inclusion of this adjuvant in our comparison. For this study, we compared the effects of the three adjuvants on the efficacy of our vaccine using a suboptimal dose (2.5 µg) of the COMC antigen, which had been determined through a previous dose response study [36], to establish whether the adjuvants ISA 61 VG and QuilA were more effective than ISA 70 VG at boosting efficacy. The suboptimal dose/adjuvant formulations were additionally compared to the benchmark 20 µg dose formulated with ISA 70 VG that had been used in all of our previous experimental vaccine trials [36,37,38].

As in the previous studies, the vaccine formulations were initially assessed via clinical outcome and compared to an unvaccinated challenge control group. Although abortions occurred in all vaccinated/challenged groups, these were only single events in the groups receiving the high (group 1) and low (group 2) COMC doses adjuvanted with ISA 70 VG, while three abortions (8%) occurred in the ISA 61 VG group (group 3). The QuilA-adjuvanted group was the least protected from the *C. abortus* challenge, with a total of nine (23.7%) ewes aborting, which was approaching the number of abortions (n = 14; 36.8%) occurring in the challenge control group (group 5). The abortion event occurring in group 1 comprised three dead lambs, which was surprising to us when this did not occur in the first two vaccine trials [37,38]. We have been unable to definitively explain this, but it appears to be linked to the response of that ewe to vaccination. We speculated in our third vaccine trial, where this also occurred [36], that it might have been related to the younger animals used than those used in the first two studies, presenting age-related differences in immune function [61]. Indeed, age and breed have been shown in other studies to affect vaccine-induced immune responses and protection [45,62,63]. We also speculate that it could be due to other factors, including stress, underlying clinical issues, the animal not being immunologically primed to respond, as suggested by the lack of any humoral response to the vaccination, as well as genetics impacting bacterial resistance and host immunity [64]. Indeed, we can see that for this particular animal, there was no measurable response to vaccination, which contrasted with the good response we observed in the lambed animals. Overall, there was little statistical difference between the three groups receiving the Montanide adjuvants, but there was a statistically significant difference between these three and the QuilA group, while the QuilA group was not statistically distinguishable from the challenge control group. This suggests that the Montanide-adjuvanted vaccine formulations were more effective in controlling *C. abortus* infection and, thus, in reducing the number of abortions in the sheep. Similarly, we do not know the reason why the three animals in groups 1 and 2 died, for which there was no pathological evidence of OEA or any other abortifacient agent; again, it could be due to any number of non-infectious reasons, including stress or any underlying subclinical issues.

In addition to clinical outcome, we also considered other factors, which impact the potential for transmission of the pathogen following parturition, in assessing the efficacy of each vaccine formulation. These factors included placental gross pathology (measured by determining the extent of lesion coverage on the placental surface, expressed as a percentage), the identification of the pathogen in these placental lesions and fetal tissues (measured with mZN staining of placental smears and IHC of fetal tissues), and the load of *C. abortus* detected in the birth canal following parturition (measured via qPCR of vaginal swabs). Examination of the placentas from all the aborted animals revealed extensive lesions covering most of the placental surface (generally 50–100%), although we did note that three placentas were recorded as having low coverage (15–25%), which is unusual but is something we have occasionally previously observed, although here, we cannot discount a possible error in recording the figures by the evaluator. In all these cases, pathological examination of the lesions confirmed all placental pathologies to be consistent with OEA. In terms of the placentas from animals that successfully lambed, we noted a small number in groups 1 and 2 (one and four placentas, respectively) that generally had a low level (1–10%) of visible gross lesion coverage. Interestingly, despite having a couple of extra abortions, which might have suggested that more lambed placentas would have lesions, none of the group 3 placentas exhibited any gross lesions. In contrast to the Montanide-adjuvanted groups (groups 1–3), the placentas of the lambed animals in group 4 that received the QuilA vaccine formulation exhibited a large increase in the number of positives (15 of 28; 53.6%), similar to what was also found in the challenge control group (14 of 23; 60.9%), again showing evidence that the QuilA-adjuvanted vaccine was not as efficacious and protective as the Montanide-adjuvanted groups in reducing the incidence of abortion. Indeed, it was evident that the QuilA-adjuvanted COMC vaccine provided no statistically significant protection. The placental smear results were essentially the same as for the gross pathology, revealing a few (n = 4) of the group 3 placentas as positive and an additional couple of positive placentas in groups 4 and 5.

The vaginal swab qPCR data revealed a large increase in the number of positive lambed animals across all challenged groups (groups 1–5). Overall, groups 1–3 had the lowest proportion of qPCR-positive animals, with group 1 having the lowest proportion (38.9%), followed by group 3 (47.1%) and then group 2 (66.7%), suggesting that the 2.5 µg dose of COMC adjuvanted with ISA 61 VG was perhaps more effective at reducing infectivity compared to when it was adjuvanted with ISA 70 VG. However, we need to also take into account the fact that there was a lower number of abortions in group 2 compared to group 3; plus, we also noted that in this trial, the group 2 66.7% infectivity rate in the lambed animals was considerably better than the 88.9% we observed in our previous study, although the reason for this is not clear. Furthermore, despite the infectivity levels observed in the lambed animals in these three groups, it is clear from the qPCR data that the number of genome copies present was very low, as evidenced by the low geometric means (range: 107–240 genome copies), when compared to the aborted animals (geometric mean: 3.2 × 10^6^ genome copies; range: 5.4 × 10^5^–1.05 × 10^7^ copies). In contrast to these results, the group 4 animals had a much higher bacterial load in the lambed animals, as reflected in the considerably higher geometric mean (4.1 × 10^4^ genome copies), which was in keeping with what was observed in the lambed animals in the challenge control group (5.8 × 10^4^ genome copies), showing that these animals would have a much greater impact on their potential for transmission to uninfected naïve animals. So, overall, taking all the clinical and bacteriology findings into consideration, we can safely say that the Montanide-adjuvanted vaccines were much more effective at reducing infectivity, abortion, and the level of shed organisms at parturition when compared to the QuilA-adjuvanted group. Thus, vaccinated animals would not only be protected against disease but would also pose a lower risk to susceptible animals through reduced bacterial shedding.

The abortions that occurred in the four vaccinated groups were all found to have high bacterial loads (range: 5.3 × 10^4^–1.4 × 10^7^), comparable to those found in the challenge control group (range: 6.0 × 10^5^–1.2 × 10^7^), as well as those we have observed in previous studies [36,37,38,40]. We do not know why these vaccinated animals aborted, but it could be due to a number of possible reasons, as we have discussed above. But, interestingly, when we look at the antibody responses to vaccination for these animals that aborted, we can see that there was very little, if any, response in groups 1–3, in contrast to the much higher response that was observed for the group 4 animals. This supports the view that antibodies have little role to play in terms of a protective response to infection [65,66]. Indeed, while group 1 does show a greater response to vaccination in animals that ultimately lambed compared to the one that aborted (which could be construed as a protective effect), in groups 2 and 3, the responses in both lambed and aborted animals were fairly comparable and very low, only increasing just prior to challenge. Therefore, the responses in the Montanide vaccinated groups are perhaps just reflective of the difference in the administered dose (20 versus 2.5 µg). If this is the case, then it is clear that QuilA elicits a stronger humoral response, which is not protective following challenge.

The antibody profiles of the three Montanide vaccinated groups (groups 1–3) post-challenge and post-parturition were not statistically distinguishable and somewhat similar to the challenge control group in that the aborted animal responses were greater than in the lambed animals. This difference in response has been observed in our previous studies [37,38], again agreeing with antibodies having little or no role in establishing placental infection but instead being the result of the greater antigenic stimulation that occurs following the rapid increase in *C. abortus* in the placentas of non-protected animals around parturition. The group 4 animals had elevated antibody titers post-challenge that were not distinguishable by outcome, providing further evidence that the magnitude of the antibody response does not correlate with protection. In contrast, cellular responses have been suggested to have a much greater role than antibody in controlling infections due to *C. abortus* [67,68], in particular the pro-inflammatory cytokine IFN-γ, which is known to restrict the growth of the pathogen [68,69]. Another pro-inflammatory cytokine, IL-17A, which is secreted by Th-17 CD4+ T cells, is involved in the host defense against microbial organisms, but its role in protecting ruminants from chlamydial infections is currently unknown [70,71]. Thus, in this study, we undertook measuring IL-17A as well as IFN-γ production at specific key points during pregnancy, specifically following vaccination, challenge, and during parturition.

Initially, following vaccination, COMC-driven IFN-γ responses were generally higher than UV-inactivated-EB-driven responses across all vaccinated groups but evened out following experimental challenge. This phenomenon is consistent with COMC vaccine-induced immune priming, where, prior to challenge, the animal’s immune systems were only exposed to the antigens present in the COMC preparation, whereas post-challenge, they were exposed to additional antigens present in the whole EB. However, it is also possible that the UV inactivation altered the antigens on the surface of the EB, which made them less immunogenic. It should be noted that the relative abundance and immunogenicity of the antigens are likely to be unequal. These IFN-γ responses also tied in with the pregnancy outcome, with the lambed animals having higher responses than those that aborted in the subsequent sample points assessed in this study, thus corresponding with an increased protective effect. When comparing the standard (group 1; 20 µg) and suboptimal (group 2; 2.5 µg) dose groups, the higher antigen dose stimulated stronger responses in the protected (lambed subgroup) animals, evident at the initial post-vaccination bleeds, prior to challenge. Direct comparison of the Montanide adjuvant groups suggests ISA 61 VG could be more effective purely based on the COMC-stimulated IFN-γ response, but the 8.1% abortion rate affects the view of its overall protective capacity. Assessing the Montanide groups together, the overall protective efficacy is more nuanced to untangle which groups have the best protective efficacy. Groups 1 and 2 have the lowest abortion rates, but group 3 has the lowest gross pathology percentage lesion coverage and lower infection rate when compared with the other suboptimal vaccine antigen dose group 2. The QuilA group 4 stimulated slighter lower vaccine-induced, COMC-specific IFN-γ alongside a high abortion rate of 23.7% and much higher infection rate when compared with the Montanide groups.

The effectiveness of IFN-γ is influenced by the entire cocktail of cytokines produced concurrently by the primed CD4+ T cells, including IL-17A, IL-10, and IL-4. Following the recent development of tools to measure ovine IL-17A [70], we were able to apply these to this investigation. A recent study investigating vaccine-induced protection to *C. trachomatis* in an experimental rodent model [72] showed that CD4+ T-cell IL-17A was partially protective in mice deficient in IFN-γ, while other animal models for chlamydial disease suggest a role for IL-17 in host defense against intracellular bacterial infection [73,74]. In this study, the Montanide-adjuvanted vaccine formulations were primed for elevated IL-17A responses post-challenge compared to QuilA and the non-vaccinated challenge group. However, there was no difference in the responses observed between animals that lambed or aborted, suggesting that in this vaccine model, IL-17A is not a strong correlate for protection, as has been observed in other studies [75,76]. Thus, the functional significance of IL-17A for protection against OEA remains to be determined in more detail. In contrast, none of the vaccine formulations induced elevated levels of antigen-specific IL-4 or IL-10. Taken together, the Montanide formulations that induced the best protection against abortion and reduced bacterial shedding also primed for chlamydial antigen-specific inflammatory cytokines (IFN-γ and IL-17A) but not regulatory (IL-4) or anti-inflammatory (IL-10) cytokines, fitting with the paradigm that vaccines capable of inducing Th-1 type responses are likely to be protective against chlamydial infections.

## 5. Conclusions

This is the last in a series of studies that have developed and optimized a new COMC vaccine to protect sheep from OEA. Previous studies have shown that the vaccine can be delivered as a single inoculation at a dose as low as 7 µg antigen when combined with the adjuvant Montanide ISA 70 VG. In this study, the effects of an additional two adjuvants, Montanide ISA 61 VG and QuilA, on vaccine efficacy were compared using a suboptimal 2.5 µg dose of the antigen against the benchmark 20 µg dose utilized in the previous studies [36,37,38]. Through a series of investigations evaluating the clinical outcome at parturition, assessment of gross placental pathology, pathogen load, assessment of shedding post-parturition, and assessment of serological and cellular responses to the vaccination and challenge, we did not see much difference between the ISA 61 VG- and ISA 70 VG-formulated COMC vaccines. Both adjuvants elicited very good efficacy and improved on the previous suboptimal assessment of the COMC vaccine, although they were not as effective as the benchmark 20 µg dose, as expected. In contrast, the QuilA adjuvant resulted in a much higher abortion rate, had a much greater overall bacterial load, and had a large increased potential risk in terms of transmission of infection to other animals and, therefore, would not be a suitable adjuvant for use in the COMC vaccine formulation. We suggested in our previous study that a dose of around 10 µg would be optimal for maximizing efficacy, while this study suggests that the vaccine could be formulated with either of the Montanide adjuvants, and this will now be taken forward for commercial consideration and any further potential refinement necessary to achieve market authorization.

## Figures and Tables

**Table 1:** The clinical outcome of pregnancy in vaccinated ewes that were challenged with *Chlamydia abortus* strain S26/3 at day 70 of gestation (groups 1–4), non-vaccinated challenge control ewes (group 5), and uninfected non-vaccinated negative control ewes (group 6).

Group ^1^	Ewes			Average Length of Gestation		Number of Lambs	
(Dose/ Adjuvant)	No. Pregnant	No. Lambed (%)	No. Aborted (%)	Lambed	Aborted	Viable	Dead
1 (20/70VG)	37	36 (97.3)	1 (2.7)	146.5	135.0	60	5 ^2^
2 (2.5/70VG)	31	30 (96.8)	1 (3.2)	146.1	140.0	45	2 ^3^
3 (2.5/61VG)	37	34 (91.9)	3 (8.1)	146.1	136.0	58	5
4 (2.5/QuilA)	38	29 (76.3)	9 (23.7)	146.0	132.7	47	13
5	38	24 (63.2)	14 (36.8)	144.7	134.7	41	21
6	7	7 (100)	0 (0)	146.9	-	14	0

^1^ Groups 1–4, vaccinated dose of COMC in μg and adjuvant used (ISA 70 VG, ISA 61VG, and QuilA) is indicated in brackets; group 5, challenge controls; and group 6, negative controls. ^2^ Includes the three dead lambs as a result of OEA from a single ewe, and one lamb found dead from each of two other ewes, which, following bacteriological and pathological investigations, were found not to be due to OEA. ^3^ Includes the single dead lamb as a result of OEA, and the delivery of a tiny, mummified fetus with no bacteriological or pathological evidence of OEA. No neonatal deaths were observed.

**Table 2:** Detection of pathological changes, *C. abortus* organisms, and genomic DNA in the placentas and vaginal swabs of vaccinated ewes that were challenged with *C. abortus* at day 70 of gestation (groups 1–4) and in infected (group 5) and uninfected (group 6) control ewes.

Group ^1^ (Dose/ Adjuvant)	Pregnancy Outcome	No. Ewes	Lesions ^2^	Smears ^3^	Swab qPCR ^4^	Swab qPCR Load ^5^
1 (20/70VG)	Lambed	36 ^7^	1+, 34	1+, 34−	14+, 22−	107 (1.33)
	Aborted	1	1+	1+	1+	n/a ^6^
2 (2.5/70VG)	Lambed	30	4+, 26−	4+, 26−	20+, 10−	240 (1.63)
	Aborted	1	1+	1+	1+	n/a ^6^
3 (2.5/61VG)	Lambed	34	0+, 34−	4+, 30−	16+, 18−	196 (1.51)
	Aborted	3	3+	3+	3+	3,211,854 (2.48)
4 (2.5/QuilA)	Lambed	29 ^7^	15+, 13−	17+, 11−	27+, 2−	41,402 (1.99)
	Aborted	9 ^7^	8+, 0−	8+, 0−	9+, 0−	1,070,840 (1.84)
5	Lambed	24 ^7^	14+, 9−	16+, 7−	23+, 1−	58,283 (2.15)
	Aborted	14	13+, 1−	13+, 1−	14+, 0−	2,848,149 (1.32)
6	Lambed	7	7−	7−	7−	13 (1.87)

^1^ Groups 1–4, vaccinated dose of COMC (in μg) and adjuvant used (ISA 70 VG, ISA 61VG, and QuilA) is indicated in brackets; group 5, challenge controls; and group 6, negative controls. ^2^ Number of ewes with gross pathological lesions characteristic of *C. abortus* infection evident in one or more placentas: +, positive; −, negative. ^3^ Number of ewes with chlamydial organisms detected following modified Ziehl–Neelsen (mZN) staining of placental impression smears: +, positive; − negative. ^4^ Number of ewes with chlamydial organisms detected on vaginal swabs via quantitative real-time polymerase chain reaction (qPCR): +, positive; −, negative. ^5^ Geometric mean (geometric SEM) of the number of *C. abortus* genomes per 1 µL of total DNA extracted from vaginal swabs and detected via qPCR. ^6^ n/a, not applicable (summary statistics cannot be computed for a single value). ^7^ No placentas were recovered from a single animal in each of the indicated groups (also see Table S1).

## Data Availability

Data are contained within the article or Supplementary Materials. The original contributions presented in the study are included in the article/Supplementary Materials, and further inquiries can be directed to the corresponding author.

## Supplementary Materials

The following supporting information can be downloaded at https://www.mdpi.com/article/10.3390/vaccines13060609/s1: Table S1: Raw data on clinical outcome, placental gross pathology, mZN, and PCR analysis for each experimental group; Table S2: Raw data for serological and cellular analyses.
